# Evaluation of recombinase polymerase amplification assays for targeted detection of bovine respiratory disease bacterial pathogens and antimicrobial-resistance genes in feedlot calves

**DOI:** 10.1177/10406387261423941

**Published:** 2026-03-04

**Authors:** Tara Funk, Lianne McLeod, Rahat Zaheer, Curtis Claassen, Christina Yevtushenko, Cheyenne Conrad, Jennifer Abi Younes, Morgan Lehmann, Sheryl Gow, Bruce Wobeser, Simon J. G. Otto, Cheryl Waldner, Tim McAllister

**Affiliations:** Western College of Veterinary Medicine, University of Saskatchewan, Saskatoon, Canada; Western College of Veterinary Medicine, University of Saskatchewan, Saskatoon, Canada; Lethbridge Research and Development Centre, Agriculture and Agri-Food Canada, Lethbridge, Canada; Lethbridge Research and Development Centre, Agriculture and Agri-Food Canada, Lethbridge, Canada; Lethbridge Research and Development Centre, Agriculture and Agri-Food Canada, Lethbridge, Canada; Lethbridge Research and Development Centre, Agriculture and Agri-Food Canada, Lethbridge, Canada; Western College of Veterinary Medicine, University of Saskatchewan, Saskatoon, Canada; Western College of Veterinary Medicine, University of Saskatchewan, Saskatoon, Canada; Canadian Integrated Program for Antimicrobial Resistance Surveillance, Public Health Agency of Canada, Saskatoon, Canada; Western College of Veterinary Medicine, University of Saskatchewan, Saskatoon, Canada; Human-Environment-Animal Transdisciplinary AMR Research Group, School of Public Health, University of Alberta, Edmonton, Canada; Western College of Veterinary Medicine, University of Saskatchewan, Saskatoon, Canada; Lethbridge Research and Development Centre, Agriculture and Agri-Food Canada, Lethbridge, Canada

**Keywords:** Bayesian latent class modeling, bovine respiratory disease, *Histophilus somni*, integrative and conjugative elements, macrolide-resistance genes, *Mannheimia haemolytica*, *Pasteurella multocida*, recombinase polymerase amplification

## Abstract

Antimicrobial resistance (AMR) challenges the effective treatment of bovine respiratory disease (BRD). We evaluated the performance of a recombinase polymerase amplification (RPA) assay, a rapid, isothermal nucleic-acid amplification method, compared with bacterial culture (BC), antimicrobial susceptibility testing (AST), and real-time PCR (rtPCR) testing. We cultured deep nasopharyngeal swabs collected from 800 beef calves within 36 d on feed and at first treatment for BRD for *Mannheimia haemolytica, Pasteurella multocida*, and *Histophilus somni*, and screened for these species and *Mycoplasmopsis bovis* using RPA (*M. haemolytica* serotypes 1 and 6 only) and rtPCR (*M. bovis* only). We then tested samples that were RPA-positive for *Pasteurellaceae* for integrative and conjugative element (ICE) variants containing *tetH* (ICE*tnpA*, ICE*ebrB*) and macrolide antimicrobial-resistance genes (ARGs; *msrE-mphE*, *erm42*). Bayesian latent class models estimated the clinical sensitivity of BC to be higher than RPA for *Pasteurellaceae* detection. Both methods were highly specific. RPA sensitivity for *M. bovis* detection was comparable to rtPCR, but RPA specificity was higher. RPA specificity for detection of macrolide resistance was lower (93.5%) than BC-AST (99.9%), reflecting the identification of ARGs by RPA in non-target bacteria. However, the sensitivity of both tests was low (BC-AST: 20.5%; RPA: 13.3%). Limited RPA sensitivity for *Pasteurellaceae* identification constrained its downstream performance for detecting ARGs. With our large-scale study, we demonstrated that RPA could detect key BRD-associated pathogens and AMR determinants directly from respiratory samples. Although our RPA results were not sufficient to inform AMU treatment strategies, RPA testing could prove valuable for addressing focused investigations with rapid turnaround.

Bovine respiratory disease (**BRD**) and liver abscesses are the primary drivers of antimicrobial use (**AMU**) in Canadian feedlot cattle.^16^ However, the emergence of antimicrobial resistance (**AMR**) threatens the continued efficacy of antimicrobial drugs used to manage these and other diseases.^56^ Several medically important antimicrobials, including macrolides and tetracyclines, are among the most frequently used classes for the prevention of liver abscesses and management of BRD.^16^

The World Health Organization has issued international guidelines promoting prudent AMU in livestock to support antimicrobial stewardship and limit the transmission of AMR.^66^ These guidelines recommend restricting the use of medically important antimicrobials in food-producing animals, especially in the absence of current laboratory evidence to inform treatment decisions.^4^ Given that antimicrobials are considered instrumental for managing the risk of BRD in feedlot production,^39^ the beef industry is under increasing pressure to examine, and where feasible, reduce AMU.^4^

The multifactorial nature of BRD comprises several risk factors for calves entering feedlots, including abrupt weaning or a lack of preconditioning, commingling and purchase from auction markets, and viral infections; these contribute to immunosuppression and predispose cattle to disease.^36,64,65^ *Mannheimia haemolytica, Pasteurella multocida, Histophilus somni*, and *Mycoplasmopsis bovis* are the bacteria associated most frequently with BRD.^36^

Bacterial culture (**BC**) and antimicrobial susceptibility testing (**AST**) have been considered the gold-standard laboratory methods for detection of BRD-related bacterial pathogens and AMR.^8^ However, detection of all organisms of interest in deep nasopharyngeal swabs (**DNPSs**) can be limited by competitive inhibition, specialized growth requirements, dynamic bacterial shedding patterns, or limited sampling within a calf’s nasal cavity.^19,37^ Recovered colonies are then subject to AST, with resulting phenotypic data potentially informing AMU protocols. However, this testing strategy is time-consuming and resource-intensive, which might not always be suitable for making timely clinical decisions regarding AMU.^25^ For *M. bovis*, rtPCR is typically preferred given the rapid turnaround time and lower cost to identify this fastidious species.^6,17^

The development of a rapid test to support AMU for BRD treatment is a priority for the beef industry.^35^ Recombinase polymerase amplification (**RPA**) is a novel laboratory method for the experimental detection of DNA-based targets that could potentially complement BC and AST as a point-of-care laboratory test. With minimal equipment requirements, RPA reactions occur at an isothermal temperature of 37–42°C and can achieve results in ≤30 min from extracted DNA.^24,25,32^ To date, RPA assays have been established for detection of bacterial and viral pathogens, mobile genetic elements, and antimicrobial-resistance genes (**ARGs**) relevant to BRD.^24,25,32,40,67,68^ However, their utility as high-throughput laboratory tools remains untested.^24,25,40,68^

Although several veterinary scientists have investigated the disease or resistance characteristics of BRD-associated bacteria isolated from moribund or dead cattle,^9,15,46,47,62^ disease onset typically occurs within the first 100 DOF.^10,36,41^ We designed a study to test multiple RPA assays for detecting BRD-related targets in a large cohort of fall-placed calves sourced from an auction market, a known predisposing factor for BRD.^52,64^ Assessing AMR prevalence in a subset of calves from an individual feedlot pen at and shortly after arrival could allow veterinarians to make informed decisions on AMU.

We aimed to apply established RPA assays to determine the prevalence of key bacterial BRD pathogens and frequently observed AMR determinants of interest, including genetic sequences linked to integrative and conjugative elements (**ICEs**), in DNPSs collected from recently arrived, fall-placed feedlot calves. ICEs facilitate the excision, replication, and horizontal transfer of accessory genes, playing a major role in the dissemination of ARGs.^31,44^ Using Bayesian latent class models (**BLCMs**), our second objective was to estimate the clinical sensitivity and specificity of RPA for each target compared with BC, AST, or real-time PCR (rtPCR) assays available in veterinary diagnostic laboratories. All assays were interpreted at the sample level, as they would be for routine laboratory testing (i.e., the presence or absence of the target of interest).

## Materials and methods

### Sample population

The sampling protocol and procedures for our project have been described in detail elsewhere^1^ and were approved by the University Animal Care Committee (Animal Use Protocol 20190069; University of Saskatchewan, Saskatoon, SK, Canada). Briefly, 100 recently weaned, fall-placed steer calves at risk for BRD were purchased at auction each week for 8 wk (*n* = 800), from 2021 October–December, and placed at the Livestock and Forage Centre of Excellence (LFCE) feedlot (University of Saskatchewan, Clavet, SK, Canada).

### Calf processing and management

As described previously,^1^ the steer calves arrived at the feedlot in the late evening and were rested in a holding pen with feed and water until the next morning, when they were weighed and processed at 1 d on feed (1 DOF) using protocols typical for moderate- to high-risk calves in commercial feedlots. The mean weight on arrival was 225 kg (range: 160–315 kg; SD: 15 kg). Processing at 1 DOF included the placement of an identification ear tag, verification of castration, and SC administration of a modified live viral vaccine that included a *M. haemolytica* toxoid (Pyramid 5 + Presponse; Boehringer Ingelheim) and a multivalent clostridial vaccine (Ultrachoice8; Zoetis). Metaphylactic oxytetracycline (OTC; Oxyvet 200 LA, Vetoquinol; 20 mg/kg) was administered by SC injection to 400 steers assigned to 4 pens; metaphylactic tulathromycin (TUL; Draxxin, Zoetis; 2.5 mg/kg) was administered by injection to 400 steers assigned to the remaining 4 pens. All calves received a growth implant ([zeranol] Ralgro; Merck) and were treated topically with an anthelmintic (Solmectin; Solvet).

Following processing, calves were housed in groups of 100 (8 pens × 100 calves) in outdoor, dirt-floor pens for the duration of the 45-d study. At 1 DOF, calves were fed a high-forage starter ration to encourage consumption, but switched to a diet of 59% barley silage, 20% hay, 15% barley grain, and 6% canola meal for the remainder of the feeding period.^1^ The diet was also supplemented with salt and vitamins A, D, and E, as well as monensin concentrate (33 mg/kg dry matter).^1^

### Sampling procedures

As described in our previous study,^1^ all calves were sampled with DNPSs at 1 DOF, before the administration of metaphylactic antimicrobials and then again at 13 DOF. Two calves died before the second sample collection. In addition, a random subset of 30 calves from each pen was sampled at 36 DOF, except for pen 8, where an outbreak of *H. somni–*associated BRD occurred before 36 DOF. In pen 8, 20 calves were sampled randomly at 30 DOF, when all calves in the pen were subsequently treated with injectable oxytetracycline (Oxyvet 200 LA; 20 mg/kg). At 36 DOF, 20 additional calves were sampled after treatment. Last, 102 DNPS samples were also collected before therapeutic antimicrobial treatment from all calves that met the case definition for BRD.^1^ Calves with signs of respiratory disease were identified by experienced feedlot personnel, using a clinical scoring system (DART—depression, appetite, respiratory signs, temperature) to diagnose BRD and gauge the severity of disease.^63^ A standard numerical scale was used to grade the severity of clinical signs from 0 (clinically normal) to 4 (moribund). Our BRD case definition required a DART score of 1 or 2 and a rectal temperature of ≥40°C, or a score of 3 or 4, regardless of temperature, as well as no other obvious potential causes of illness.^1^ We collected 1,950 samples across all times from the 800 calves in our study.

As described previously,^1^ at each sampling time, calves were restrained in a hydraulic chute using a neck extender, and 3 DNPSs were collected. A single-use paper towel was used to wipe the external nares, and a double-guarded culture swab (Continental Plastic) was directed into the ventral meatus of the nostril. The polyester-tipped swab was advanced through the inner sheath and vigorously rotated against the nasopharyngeal mucosa 5–6 times. The swab was withdrawn into the inner sheath and outer guard before removal from the nostril. Approximately 3 cm of swab tip was cut and placed in a 15-mL vial containing 3 mL of liquid Amies transport medium (University of Saskatchewan). Two additional samples were obtained from alternating nostrils using the same procedure, and all 3 DNPSs per calf were pooled in the same vial.

Samples collected at the planned times were immediately transported in coolers with icepacks to the laboratory (Western College of Veterinary Medicine, University of Saskatchewan) for same-day processing. Samples collected from calves at first treatment for BRD were stored at the LFCE feedlot and refrigerated for up to 72 h before they were transported on ice to the laboratory for processing. Each pooled sample vial was vortexed for 1 min, and the sample suspension was aliquoted for experimental testing.

### Experimental testing of deep nasopharyngeal swab samples

#### Sample testing strategy

Collected swabs were subject to 3 test methods described in subsequent sections: 1) RPA, 2) BC and AST, or 3) rtPCR. Our testing strategy for the identification of BRD-associated bacterial pathogens included testing for *M. haemolytica*, *P. multocida*, *H. somni* by RPA and BC, and for *M. bovis* using RPA and rtPCR. Only samples harboring *M. haemolytica*, *P. multocida*, or *H. somni* detected by RPA were tested for ICEs and macrolide ARGs using RPA. The results of macrolide ARG detection were compared with AST data on phenotypic macrolide resistance.

#### DNA extraction

Before RPA testing, a 1.0-mL aliquot of vortexed, raw sample suspension was added to a standard 1.5-mL Eppendorf tube, and DNA was extracted (DNeasy; Qiagen), following the manufacturer’s protocol. Each tube was centrifuged for 6 min at 5,000 × *g* and 20°C. The supernatant was discarded, and the pellet was resuspended in 200 µL of PBS. Cells were lysed by adding 200 µL of tissue lysis buffer (Buffer ATL; Qiagen) and 20 µL of proteinase K, followed by incubation at 56°C for 30 min on a shaker plate set to 300 rpm.

Sample tubes were mixed by inversion following the addition of 200 µL of 95% ethanol. Two wash steps (Buffers AW1 and AW2; Qiagen) were completed using a motorized vacuum manifold. Extracted DNA was eluted in 100 µL (Buffer AE; Qiagen) and stored at 4°C until further testing. DNA concentration was measured for all samples (Qubit fluorometer, Qubit dsDNA BR assay kit; ThermoFisher).

Positive control template DNA required for RPA testing was generated for *M. haemolytica*, *P. multocida*, *H. somni*, 2 ICE variants, and 3 clinically relevant macrolide-resistance genes, according to established protocols.^24,25,32^

#### Recombinase polymerase amplification

Extracted DNA was available for RPA testing from 1,153 samples, 414 at collected at 1 DOF, 415 at 13 DOF, 250 at 36 DOF, and from 74 calves at first treatment for BRD. Samples available for testing included 74 of 102 sick calves, all 250 calves tested at 36 DOF, and samples collected from 323 calves at 1 DOF and 324 at 13 DOF that were also sampled later at 36 DOF or as sick calves. To achieve at least 50 samples tested from each of the 8 pens at 1 DOF and 13 DOF, matching sample pairs collected from 91 calves at both 1 DOF and 13 DOF were randomly selected for RPA testing.

Multiplex, real-time RPA assays for bacteria and ICEs were completed at 37°C for 33 min on a T16-ISO machine (Axxin, Australia), using a 50-µL reaction volume in 0.2-mL strip tubes.^24,25^ A fluorescence threshold of ≥100 mV for ≥60 s was used to define a “positive” fluorescence signal for all 3 multiplex reactions (*M. haemolytica*/*M. bovis*, *P. multocida*/*H. somni*, ICE*tnpA*/ICE*ebrB*).^25^ Reactions targeting *msrE-mphE*/*erm42* ran for 26 min at 39°C, with a fluorescence threshold of ≥400 mV for ≥60 s.^32^ The T16-ISO RPA machine contained 16 tube spaces, of which 2 were allocated for an negative template control and positive control in each reaction, leaving 14 spaces for testing extracted sample DNA.

**Figure 1.**
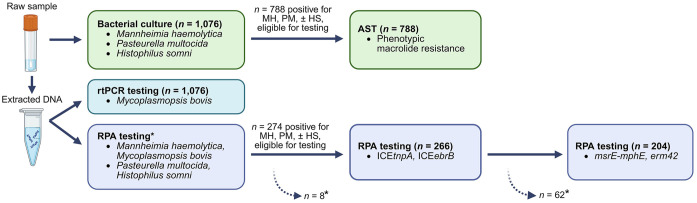
Flowchart of the testing strategy used for bacterial culture, antimicrobial susceptibility testing (AST), real-time PCR (rtPCR), and recombinase polymerase amplification (RPA) of deep nasopharyngeal swabs collected from feedlot calves at arrival, or days 13 or 36 on feed. HS = *Histophilus somni*; ICE = integrative and conjugative element; MH = *Mannheimia haemolytica*; PM = *Pasteurella multocida*. *Two samples did not contain sufficient volume for *P. multocida* and *H. somni* RPA testing (*n* = 1,074). Of the 274 swab samples that were RPA-positive for *M. haemolytica*, *P. multocida*, and/or *H. somni*, 8 samples did not contain sufficient volume for further testing for ICE variants ICE*tnpA* and ICE*ebrB.* Following RPA testing for ICEs, a further 62 samples were volume insufficient and were not tested for macrolide-resistance genes *msrE-mphE* and *erm42* (*n* = 204). Note: To calculate population-level prevalence, assuming a constant prevalence of the RPA target of interest (ICEs or macrolide-resistance genes), the numerator was corrected to include the expected positive results from the samples with insufficient volume.

We used RPA assays to detect *M. haemolytica* serotypes 1 and 6, *P. multocida*, *H. somni*, and *M. bovis*,^24^ ICE variants (ICE*tnpA: tetH_tnpA*; ICE*ebrB: tetH_ebrB*),^25^ and macrolide ARGs (*msrE-mphE* operon, *erm42*; **Table 1**).^32^ Reactions were run in the following multiplex formats: *M. haemolytica*/*M. bovis*, *P. multocida*/*H. somni*,^24^ ICE*tnpA*/ICE*ebrB*,^25^ and *msrE-mphE*/*erm42*^32^ (**Fig. 1**). Given this testing strategy, each sample was subjected to 2 RPA assays for detection of the bacterial BRD targets (2 × multiplex assays), with an additional 2 assays for ICE variants (1 × multiplex assay) and macrolide ARGs (1 × multiplex assay) if a sample was positive for *M. haemolytica*, *P. multocida*, or *H. somni*.

**Table 1.** Summary of primers and probes used to identify bacterial pathogens, integrative and conjugative elements (ICEs), and macrolide-resistance genes (ARGs) in deep nasopharyngeal swabs collected from feedlot calves.

Target	Gene	Forward primer sequence	Reverse primer sequence	Amplicon size, bp	Fluorescence probe sequence*	Assay type	Ref.
Bacterial pathogens associated with bovine respiratory disease							
*Histophilus somni*	Hs_0116	CGTTTAATCCCATTGCGATCATTCCCCATT	ATACTATTGCATTCGGCGATTTTTCCGCTT	342	TATTCAAGTAGATGCAGATGGGCAGCATAAFHQAATTGATGTCAAGAA	Hs/Pm multiplex	^25^
*Mannheimia haemolytica* serotypes 1,6	*nmaA*	TCAAAATGGCTCCCTTAGTTGAGGGCTTTA	AGTGGTTGCTGTATCGCCATGAACAAAAAT	254	TTCTGCTATTTTAGAAAAAATTCAACCTGTFHQTGCCGAATACAAAC	Mh/Mb multiplex	^25^
*Mycoplasmopsis bovis*	*uvrC*	ATGGTCCTTTTCCTTCTGGTTATGGAGCTA	TGGCTGCTTGATGCATTTTGTTAGTTAGTT	201	CAAAGACTATAACTTTTGGATTAATCAGTTFHAQAAAATTAAAGAAATT	Mh Mb multiplex	^25^
*Pasteurella multocida*	*kmt1*	GAACCGATTGCCGCGAAATTGAGTTTTATG	CGAACTCGCCACTTTTTGTTTCATTTGGAC	417	ATTATTTTATGGCTCGTTGTGAGTGGGCTTGFHGGQAGTCTTTTATTT	Hs/Pm multiplex	^25^
Antimicrobial-resistance determinants							
ICE*tnpA*†	*tetH_tnpA*	CATCCACTAACTACGGCGCTGACATATCAA	TTGGTCCCCTTTTATTTGCCTTTATTTATA	318	ACACTGGTGCGGGAGATTGAGGCGGGGCGTFTHQAGCCCTGTTTCAACCC	ICE*tnpA*/ICE*ebrB* multiplex	^26^
ICE*ebrB*†	*tetH_ebrB*	GAAAAGGTCGATTTTTGGGGAATTGCGAGC	Same as *tetH_tnpA*	351	ATTGGCTTGATTTTGGCAGGTGTGATAATGAFGHAQACGCTGTCCAAAATGG	ICE*tnpA*/ICE*ebrB* multiplex	^26^
Macrolide ARGs	*msrE-mphE* operon	CGAGATCAAAGACCACAAAATCATCAAGAC	TTCTTTCTTGATTTGTTCCCTCATGCCATCAC	284	AGCCAATCTAATCCGAACATTAATTATFGAHCQCTTTAAAGGAAATTA	MM/E multiplex	^33^
Macrolide ARG	*erm42*	GGTGCACCATCTTACAAGGAGTCTTATAAATC	GCATGCCTGTCTTCAAGGTTTATATCTGTAAAGTC	176	TTTATTATATAAACCATTTTTCAAAACFAAHAQATTGCATAGCTTT	MM/E multiplex	^33^

MM/E = *msrE-mphE* and *erm42* macrolide-resistance genes.

* Within the RPA probe sequences: F = fluorophore, H = tetrahydrofuran, Q = quencher.

† A multiplex RPA assay targeted the 2 most common genetic variants of ICE containing *tetH*.

Of the 1 DOF samples, 3 had insufficient sample volume of extracted DNA for RPA testing and 2 were only tested for *M. haemolytica* and *M. bovis*, also because of insufficient extracted DNA volume (*n* = 1,076 samples tested for *M. haemolytica* and *M. bovis*, *n* = 1,074 samples tested for *P. multocida* and *H. somni*). In addition, 74 samples collected from first-pull sick calves were tested with RPA. Only samples that were RPA-positive for *M. haemolytica*, *P. multocida*, and/or *H. somni* were subsequently tested for ICEs and macrolide ARGs.

#### Bacterial culture and antimicrobial susceptibility testing

Samples were cultured for *M. haemolytica*, *P. multocida*, and *H. somni* at a commercial diagnostic laboratory (Prairie Diagnostic Services [PDS]; Saskatoon, SK, Canada) the day of collection, as described previously.^1^ Testing for *M. bovis* was completed using rtPCR with methods described below.

A 10-µL aliquot of each sample was plated onto Columbia blood agar supplemented with 5% sheep blood (ThermoFisher) and incubated for 42 h at 35°C. For the isolation of *H. somni*, samples were plated onto chocolate agar and incubated for 48 h at 35°C in a 5% CO_2_ atmosphere. To confirm species identity, colonies with morphology resembling those of the BRD pathogens of interest were selected and tested using a MALDI-TOF MS instrument (Microflex LT; Bruker) and MALDI Biotyper (Microflex LT Compass software v.1.4; Bruker).

As described previously,^1^ for each sample, a single colony of each bacterial pathogen was randomly selected to undergo AST for a variety of antimicrobials by serial broth microdilution, using a BOPO7F plate and platform (Sensititre; ThermoFisher). Clinical and Laboratory Standards Institute (CLSI) guidelines were followed using the bovine respiratory–specific minimum inhibitory concentration (MIC) breakpoint^23^ for *M. haemolytica*, *P. multocida*, and *H. somni*. Susceptibility results were collected for ampicillin (AMP), ceftiofur (TIO), danofloxacin (DANO), enrofloxacin (ENR), florfenicol (FLOR), gamithromycin (GAM), penicillin (PEN), spectinomycin (SPT), tetracycline (TET), tildipirosin (TILD), tilmicosin (TIL), and TUL. Bacterial isolates were designated as susceptible, intermediate, or resistant; for binary outcomes, isolates designated as ‘intermediate’ were reclassified as “susceptible” to that specific antimicrobial. Multiclass resistance described any isolate with phenotypic resistance to ≥3 antimicrobial classes.

#### Real-time PCR testing for Mycoplasmopsis bovis

rtPCR testing for *M. bovis* was completed by our research laboratory using a specific probe for the *oppD* gene.^58^ A standard rtPCR reaction was completed (Taqman fast advanced master mix; ThermoFisher) and compared to a standard curve created from a printed control (gBlock; Integrated DNA Technologies [IDT]). Briefly, the rtPCR reaction conditions included normalizing the sample DNA concentration to 10 ng/µL. Samples were then tested in duplicate, with 2 µL of sample template DNA added to each 10-µL reaction. A 5-µL aliquot of buffer (Taqman master mix buffer; ThermoFisher) was added to each reaction, along with forward and reverse primers at a final concentration of 0.2 µM (IDT), and a Taqman hydrolysis probe (IDT). The reaction was topped up to 10 µL with nuclease-free water (ThermoFisher), and rtPCR reactions were completed (AriaMx real-time PCR system; Agilent). Reactions were hot-started for one 5-min cycle at 45°C and then a 30-s cycle at 95°C. Each reaction then underwent 40 × cycles of amplification with the following conditions: 10 s at 95°C, 20 s at 55°C, and 20 s at 72°C. A sample was considered rtPCR-positive for *M. bovis* if the Ct value was <37.

### Statistical analysis

Detection frequency of *M. haemolytica*, *P. multocida*, *H. somni*, *M. bovis*, ICE*tnpA* and ICE*ebrB*, phenotypic macrolide resistance, *msrE*-*mphE*, and *erm42* were described using the respective testing platform: 1) RPA (all genetic targets), 2) BC and AST (*M. haemolytica*, *P. multocida*, *H. somni*, phenotypic macrolide resistance), and 3) rtPCR (*M. bovis*) using a commercial software program (Stata/IC v.15.1; StataCorp).

For both ICE and macrolide ARG detection, prevalence of these determinants was calculated for “time point” and “sick calf” samples using the number of samples tested, as well as a population-corrected denominator to reflect the total samples considered by the testing strategy, including samples of insufficient volume. For population correction, samples that were not tested per the testing strategy (i.e., if no target bacteria were detected using RPA) were classified as “negative.”

For samples that were not tested because of insufficient volume (Fig. 1), the numerator used to calculate population-level prevalence was adjusted to reflect the expected value of the positives in the missing samples. To calculate the expected value, the total number of samples eligible for testing was multiplied by the proportion of samples actually tested that were positive for each outcome. The adjusted numerator reflected the number of samples that should have been tested, assuming a constant prevalence of the RPA target of interest (i.e., ICEs or macrolide ARGs). This value was used to calculate the total percentage of positive samples for the population based on the testing strategy.

### Performance of the multiplex, real-time RPA assays

In the absence of a true gold standard test to assess the validity of real-time RPA for making clinical decisions about AMU based on samples from individual calves, BLCMs were applied to assess the performance of RPA compared with other available (but imperfect) laboratory test methods. For detection of *M. haemolytica*, *P. multocida*, and *H. somni*, RPA and BC were compared in a series of BLCMs; rtPCR was evaluated alongside RPA for *M. bovis* identification. A separate BLCM evaluated detection of macrolide-resistance targets, including *msrE-mphE* and *erm42*, in calf respiratory swab samples that were also RPA positive for *M. haemolytica, P. multocida*, or *H. somni*, compared with phenotypic macrolide resistance using AST of samples positive for the targeted *Pasteurellaceae* bacteria.

Each BLCM compared 2 tests across 5 populations; samples collected 1) on arrival from all calves, 2) on 13 DOF or 3) 36 DOF from calves that received oxytetracycline as metaphylaxis, and 4) on 13 DOF or 5) 36 DOF from calves that received tulathromycin as metaphylaxis.

For the macrolide-resistance model, detection of phenotypic resistance in *M. haemolytica*, *P. multocida*, and/or *H. somni* to any of the tested macrolides (GAM, TIL–*M. haemolytica only, TILD, or TUL) within samples was compared with the detection of macrolide ARGs *msrE-mphE* and/or *erm42* by RPA in any samples that were also RPA-positive for these bacteria. Samples in which none of these 3 bacteria was detected by RPA were considered negative for macrolide resistance associated with the target *Pasteurellaceae*.

For all BLCMs, uninformative priors (beta [1,1]) were used to estimate the clinical sensitivity and specificity of each test. Covariance was incorporated into the model for the comparison of RPA and PCR for *M. bovis* detection, to account for potential correlation between these 2 DNA-based test methods. RPA and BC-AST were distinct laboratory methods. Hence, RPA and BC-AST were considered conditionally independent, and covariance was not modeled for the comparison of these 2 tests. Models used the “runjags” package^27^ and JAGS software in R^54,55^ (R Foundation for Statistical Computing).

All BLCMs were assessed for convergence. The potential scale reduction factor (<1.05), effective sample size (>1,000), and Monte Carlo SE as a percent of SD (<5.0%), as well as visual inspection of trace and autocorrelation plots were considered satisfactory if they were within the defined limits. Model parameters were estimated using 50,000 iterations (100,000 iterations for the *M. bovis* model) in each of 3 Monte Carlo Markov chains, after a burn-in phase of 5,000 iterations per chain. Estimated sensitivity and specificity were reported for each test as the median of the posterior distribution, along with the 95% credible intervals (95% CrI).

The Epitools^59^ data analysis platform and estimated RPA sensitivity and specificity for macrolide ARG detection were also used to calculate positive predictive value (PPV) and negative predictive value (NPV), to create a graph and visualize how PPV and NPV change with various prevalences of macrolide resistance.

Kappa (κ) was calculated between RPA and BC, RPA and AST, or RPA and rtPCR (*M. bovis* only) for detection of BRD-associated bacteria, elements of macrolide resistance (phenotypic or the presence of *msrE-mphE* and/or *erm42*), or of tetracycline resistance (phenotypic or the presence of *tetH*) as a component of tested ICE gene pairs, accounting for potential agreement attributable to chance (Stata/IC v.15.1; StataCorp). Agreement was summarized as follows: κ ≤ 0.00 = poor, 0.01–0.20 = slight, 0.21–0.40 = fair, 0.41–0.60 = moderate, 0.61–0.80 = substantial, 0.81–1.00 = near perfect.^30^

The association between the presence of variants ICE*tnpA* and/or ICE*ebrB* gene pairs, both of which contain *tetH*, and the odds of also detecting phenotypic resistance to 1) tetracyclines, 2) macrolides (GAM, TIL, TILD, or TUL), 3) any of the antimicrobial drugs tested, both including and excluding TET, or 4) multiclass resistance in the DNPS samples tested were determined using generalized estimating equations (GEE; Stata/IC v.15.1). Samples that were not tested because of insufficient volume were excluded from the analysis. The GEE models were adjusted for clustering by pen, with an exchangeable correlation structure. The models accounted for the sampling time and the type of metaphylaxis administered as fixed effects. Robust SEs were used, along with a “logit” link function and a binomial distribution.

## Results

### Detection of bacterial BRD pathogens and antimicrobial-resistance determinants

#### Samples collected from calves at arrival processing, 13 DOF, and 36 DOF

##### BRD-related bacterial pathogen detection

RPA identified *M. haemolytica* serotypes 1 and 6 in 153 of 1,076 (14%) samples; 476 of 1,076 (44%) samples were positive for *M. haemolytica* using BC (**Table 2**). The number of positive samples was also higher for BC of *P. multocida* and *H. somni* than for RPA. There was less difference between the PCR and RPA test results for *M. bovis*.

**Table 2.** Summary of bacterial pathogens important in bovine respiratory disease detected by recombinase polymerase amplification (RPA) and bacterial culture or PCR testing of deep nasopharyngeal swabs collected from feedlot calves at arrival processing, and days 13 or 36 on feed (*n* = 1,076 for *Mannheimia haemolytica* and *Mycoplasmopsis bovis; n* = 1,074 for *Pasteurella multocida* and *Histophilus somni*).

Species	Count of positive samples (%)		
	RPA	Culture	PCR
*Mannheimia haemolytica* *	153 (14.2)	476 (44.2)	NA
*Pasteurella multocida*	61 (5.7)	348 (32.4)	NA
*Histophilus somni*	127 (11.8)	223 (20.8)	NA
*Mycoplasmopsis bovis*	97 (9.0)	NA	121 (11.2)

NA = not applicable.

* The RPA assay for *M. haemolytica* specifically targeted serotypes 1 and 6.

Agreement between RPA and BC for detection of bacterial BRD pathogens was slight to fair for *M. haemolytica* (κ = 0.23; 95% CI [0.18, 0.28]), *P. multocida* (κ = 0.12; 95% CI [0.074, 0.17]), and *H. somni* (κ = 0.39; 95% CI [0.32, 0.47]). Agreement was also fair between RPA and PCR for *M. bovis* detection (κ = 0.36; 95% CI [0.27, 0.45]).

##### Tetracycline resistance and ICE detection

Of the 1,076 samples tested, 274 samples were positive for *M. haemolytica, P. multocida*, or *H. somni* and 266 were further tested for ICEs using RPA (Fig. 1). ICE*tnpA* and ICE*ebrB* were identified more frequently as individual variants than together (**Table 3**). The percentage of all samples eligible for testing that were positive for either or both ICE variant gene pairs was 6.5% and was slightly lower than the 10.4% of samples in which *Pasteurellaceae* bacteria were isolated that were resistant to tetracyclines (Table 3).

**Table 3.** Prevalence of integrative and conjugative element (ICE) variants identified by recombinase polymerase amplification (RPA) of target DNA from deep nasopharyngeal swabs that also tested positive by RPA for any of *Mannheimia haemolytica*, *Pasteurella multocida*, or *Histophilus somni* and phenotypic resistance to tetracycline based on antimicrobial susceptibility testing (AST) of bacteria of interest cultured at arrival, and days 13 or 36 on feed.

Tetracycline resistance and ICE gene combination	No. of positive samples	% positive samples, by the no. of samples tested using RPA (*n* = 266)*	Estimated no. of positive samples based on eligible samples RPA testing (*n* = 274)	% positive, by the no. of samples tested using culture (*n* = 788)	% positive samples, based on the testing strategy (*n* = 1,076)
AST results†					
Phenotypic tetracycline resistance	112	NA	NA	14.2	10.4
RPA results‡					
ICE*tnpA* only	37	13.9	38	NA	3.5
ICE*ebrB* only	27	10.2	28	NA	2.6
ICE*tnpA* and ICE*ebrB*	4	1.5	4	NA	0.4
Total ICE-positive samples	68	25.6	70	NA	6.5

CE variants include ICEtnpA (tetH_tnpA target sequence) and ICEebrB (tetH_ebrB target sequence), which both contain the tetracycline-resistance gene, *tetH*. NA = not applicable.

* Total number of samples eligible for testing with sufficient volume.

† Samples tested by AST were positive for any of *M. haemolytica*, *P. multocida*, or *H. somni*.

‡ Only samples that were RPA-positive for at least one of *M. haemolytica*, *P. multocida*, or *H. somni* were eligible for testing for ICE*tnpA* and ICE*ebrB*.

Based on RPA and AST results, 28 of 265 (11%) samples harbored one or both ICE variants containing *tetH* and contained bacteria resistant to tetracycline; 40 of 265 (15%) harbored ICE*tnpA* and/or ICE*ebrB*, but no tetracycline-resistant bacteria were detected (**Table 4**). RPA detection of ICE variants containing *tetH* and phenotypic tetracycline resistance using AST had a moderate level of agreement (κ = 0.46; 95% CI [0.33, 0.59]) in samples tested by RPA. The presence of ICEs containing *tetH* was also a strong predictor for the detection of bacteria with tetracycline resistance (OR 7.0, *p* < 0.001; **Table 5**).

**Table 4.** Corresponding counts of integrative and conjugative element variants containing *tetH* identified by recombinase polymerase amplification (RPA) and phenotypic tetracycline resistance detected by antimicrobial susceptibility testing of bacteria of interest isolated from deep nasopharyngeal swabs that tested positive for *Mannheimia haemolytica*, *Pasteurella multocida*, or *Histophilus somni* using RPA at arrival, and days 13 or 36 on feed (*n* = 265).

	Phenotypic tetracycline resistance		Total
	Yes	No	
Presence of any ICE variant* containing *tetH*			
Yes	28	40	68
No	6	191	197
Total	34	231	265†

ICE = integrative and conjugative element.

* Any ICE variant was taken as evidence of ICE variant ICE*tnpA* (containing *tetH_tnpA*) or ICE*ebrB* (containing *tetH_ebrB*).

† Total no. of samples tested with RPA with sufficient volume and matching RPA and AST results of the 274 eligible for testing.

**Table 5.** Association between integrative and conjugative elements (ICEs) detected using recombinase polymerase amplification (RPA) and phenotypic resistance to tetracycline, macrolides, any antimicrobial drug tested (including or excluding tetracycline resistance), or multiclass resistance in bacteria of interest isolated from deep nasopharyngeal swabs that also tested positive for *Mannheimia haemolytica*, *Pasteurella multocida*, or *Histophilus somni* at arrival, or days 13 or 36 on feed using recombinase polymerase amplification (*n* = 1,066).

Phenotypic resistance variant	Odds ratio*	95% CI	*p* value
Any antimicrobial drug (including TET)†	5.9	[3.6, 9.7]	**<0.001**
Any antimicrobial drug (excluding TET)	3.8	[2.3, 6.5]	**<0.001**
Tetracycline	7.0	[3.6, 13.8]	**<0.001**
Macrolides (GAM, TIL, TILD, TUL)	4.2	[2.6, 6.9]	**<0.001**
Multiclass resistance	4.6	[2.9, 7.3]	**<0.001**

* The developed generalized estimating equation models for the detection of ICEs by RPA were applied using the whole sample testing strategy (*n* = 1,066), adjusting for clustering of resistance by pen, and accounting for sampling time point and the type of metaphylaxis (OTC or TUL) as fixed effects.

† Antimicrobials included in AST included ampicillin (AMP), ceftiofur (TIO), danofloxacin (DANO), enrofloxacin (ENR), florfenicol (FLOR), gamithromycin (GAM), penicillin (PEN), spectinomycin (SPECT), tetracycline (TET), tildipirosin (TILD), tilmicosin (TIL), and tulathromycin (TUL). Note: the MIC distribution of TIL was only interpreted for isolated *M. haemolytica* colonies, as Clinical and Laboratory Standards Institute–recommended breakpoints are not available for *P. multocida* and *H. somni.* Statistically significant associations (*p* ≤ 0.05) are in bold.

##### Macrolide-resistance and antimicrobial-resistance gene detection

Following RPA testing for ICEs, 204 samples were tested for the macrolide ARGs *msrE-mphE* and *erm42* (Fig. 1). The final population prevalence—adjusted for the total number of eligible samples—was higher for detection of macrolide ARGs (11.8%) than for isolation of *Pasteurellaceae* bacteria that were resistant to macrolides (3.0%; **Table 6**).

**Table 6.** Prevalence of macrolide antimicrobial-resistance genes (ARGs) detected by recombinase polymerase amplification (RPA), and phenotypic macrolide resistance detected using antimicrobial susceptibility testing (AST) of bacteria of interest isolated from deep nasopharyngeal swabs that were also positive for *Mannheimia haemolytica*, *Pasteurella multocida*, or *Histophilus somni* at arrival, or days 13 or 36 on feed using RPA.

Macrolide-resistance variant (ARG or phenotypic)	No. of positive samples	% positive, by the no. of samples tested using RPA (*n* = 204)*	Estimated no. of positive samples based on eligible samples for RPA testing (*n* = 274)	% positive, by the no. of samples tested using culture (*n* = 788)	% positive samples, based on the testing strategy (*n* = 1,076)
AST results†					
Phenotypic macrolide resistance‡	32	NA	NA	4.1	3.0
RPA results§					
*msrE-mphE* only	80	39.2	107	NA	9.9
*erm42* only	5	2.5	7	NA	0.7
*msrE-mphE* and *erm42*	10	4.9	13	NA	1.2
Total macrolide ARG-positive samples	95	46.6	127	NA	11.8

NA = not applicable.

* Total no. of samples eligible for testing with sufficient volume.

† Samples tested by AST were positive for any of *M. haemolytica*, *P. multocida*, or *H. somni*.

‡ Phenotypic macrolide resistance was defined by resistance to gamithromycin, tilmicosin, tildipirosin, and/or tulathromycin following AST.

§ Only samples that were RPA-positive for at least one of *M. haemolytica, P. multocida*, or *H. somni* were eligible for testing for macrolide ARGs.

Corresponding RPA and AST results seldom matched (4 of 204, 2%) where samples harboring *msrE-mphE* and/or *erm42* also demonstrated phenotypic macrolide resistance (**Table 7**). Macrolide ARGs were identified by RPA; the isolates recovered were susceptible to all macrolides tested in 91 of 204 (45%) samples tested by RPA. RPA and AST demonstrated very slight agreement (κ = 0.016; 95% CI [–0.039, 0.070]) for detection of macrolide ARGs and phenotypic macrolide resistance in samples tested by RPA.

**Table 7.** Corresponding counts of macrolide-resistance genes identified by recombinase polymerase amplification (RPA), and phenotypic tetracycline resistance detected by antimicrobial susceptibility testing of bacteria of interest isolated from deep nasopharyngeal swabs that also tested positive for *Mannheimia haemolytica*, *Pasteurella multocida*, or *Histophilus somni* using RPA at arrival, day 13, or day 36 on feed (*n* = 204).

	Phenotypic macrolide resistance		Total
	Yes	No	
Presence of any macrolide-resistance gene*			
Yes	4	91	95
No	3	106	109
Total	7	197	204†

* Any ARG was taken as evidence of *msrE-mphE* and/or *erm42* by RPA.

† Of the 274 eligible for testing, 204 samples tested by RPA and with sufficient volume had matching RPA and AST results.

The presence of ICE variants was strongly associated with the detection of phenotypic macrolide resistance by AST (OR 4.2, *p* < 0.001; Table 5). If at least one ICE variant was present, the odds of detecting multiclass resistance (OR 4.6; *p* < 0.001) or phenotypic resistance to at least one of the antimicrobial drugs tested (OR 5.9; *p* < 0.001) were similarly increased (Table 5).

##### Samples collected from calves treated for bovine respiratory disease

Similar to the DNPSs collected at the scheduled times, the recovery of *M. haemolytica*, *P. multocida*, and *H. somni* from the respiratory swab samples of 74 sick calves was variable, but higher by BC than by RPA (**Suppl. Table 1**). *M. bovis* was not detected by rtPCR. A higher proportion of *tetH*-containing ICE variants was detected by RPA than tetracycline-resistant *Pasteurellaceae* bacteria isolated by BC and AST (**Suppl. Table 2**). Detection of ICEs was associated with isolation of *Pasteurellaceae* with resistance to any antimicrobial tested (including tetracycline), macrolide resistance, and multiclass resistance (**Suppl. Table 3**). However, without the inclusion of tetracycline resistance, the association between ICE detection and the isolation of *Pasteurellaceae* with resistance to the remaining antimicrobial drugs tested by AST was no longer significant (Suppl. Table 3). Finally, bacteria containing macrolide ARGs *msrE-mphE* and/or *erm42* were identified in >10× more samples by RPA than phenotypic macrolide resistance was detected using BC-AST (**Suppl. Table 4**).

### Comparison of laboratory test performance

#### Bayesian latent class models

The estimated clinical sensitivity of RPA was lower than BC for all *Pasteurellaceae* targets in samples collected early in the feeding period; the specificity of RPA and BC was similar for all *Pasteurellaceae* targets (**Table 8**). The sensitivity of RPA and PCR was similar for *M. bovis*, given that the CrIs overlapped; however, the specificity of RPA was significantly higher than that of PCR.

**Table 8.** Estimated clinical sensitivity and specificity of recombinase polymerase amplification (RPA), bacterial culture, and antimicrobial susceptibility testing (AST) for detection of respiratory bacteria, macrolide antimicrobial-resistance genes (ARGs), and phenotypic macrolide resistance in bacteria of interest isolated from deep nasopharyngeal swabs obtained from feedlot calves at 3 times early in the feeding period, using Bayesian latent class models.

ARG or bacterial species name and target test	Sensitivity, % (95% CrI)	Specificity, % (95% CrI)
Mannheimia haemolytica* (n = 1,076)		
RPA (serotypes 1, 6)	27.9 (22.5, 35.2)	96.1 (93.7, 99.3)
Bacterial culture (all serotypes)	92.8 (81.6, 99.99)	93.2 (81.8, 99.99)
*Pasteurella multocida* (*n* = 1,074)		
RPA	13.3 (9.1, 18.3)	97.7 (96.1, 99.3)
Bacterial culture	90.4 (70.7, 99.99)	93.4 (86.6, 99.99)
*Histophilus somni* (*n* = 1,074)		
RPA	40.2 (33.1, 48.0)	97.2 (95.6, 98.6)
Bacterial culture	80.0 (69.5, 89.7)	98.2 (95.9, 99.99)
*Mycoplasmopsis bovis* (*n* = 1,076)		
RPA	42.5 (26.2, 82.1)	98.7 (97.4, 99.99)
rtPCR	24.0 (13.7, 45.4)	91.5 (89.3, 93.6)
Macrolide resistance (*n* = 1,002)		
RPA (*msrE-mphE* ± *erm42*)	20.5 (13.2, 29.9)	93.5 (91.2, 95.9)
Bacterial culture and AST	13.3 (6.9, 22.0)	99.9 (99.5, 99.99)

CrI = credible interval; rtPCR = real-time PCR.

* The RPA assay for *M. haemolytica* specifically targeted serotypes 1 and 6, which are more commonly associated with disease, whereas bacterial culture and rtPCR were not serotype-specific.

A similar BLCM was used to estimate the sensitivity and specificity of RPA relative to AST for detection of macrolide-resistance genes *msrE-mphE* or *erm42* (RPA) and phenotypic macrolide resistance (AST; Table 8). The estimated sensitivity of RPA and AST were both similarly low; specificity of BC was higher than for RPA.

### Predictive value of RPA detection of macrolide-resistance genes for phenotypic macrolide resistance

**Figure 2.**
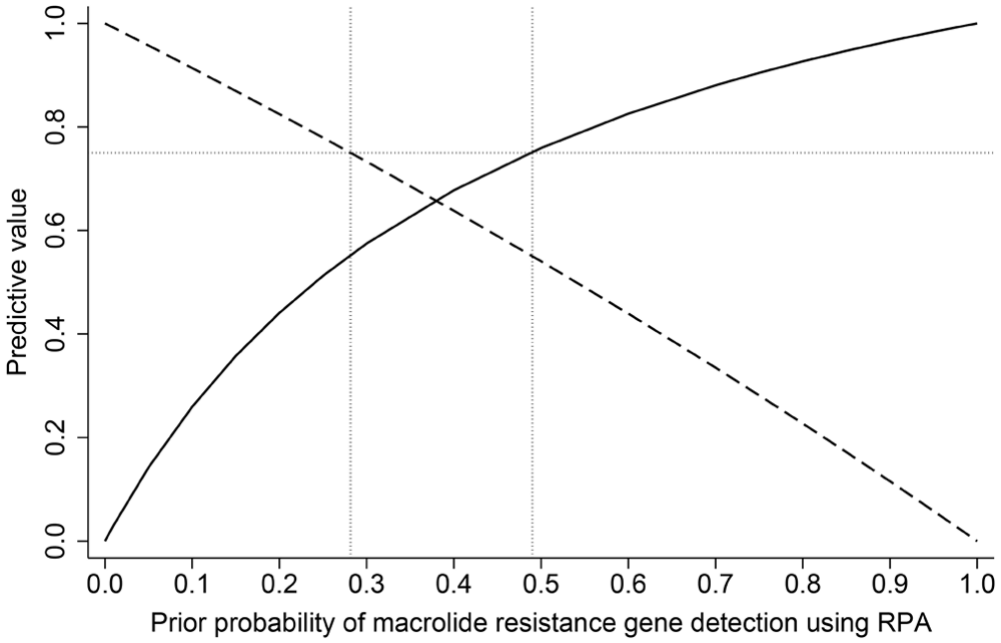
Impact of theoretical variation in the prevalence of macrolide resistance on the positive predictive value (PPV) and negative predictive value (NPV) of the recombinase polymerase amplification (RPA) testing strategy, given the clinical sensitivity (20.5%) and specificity (93.5%) estimates based on Bayesian latent class modeling. Solid line = PPV, or the probability of phenotypic macrolide resistance given the detection of *msrE-mphE* and/or *erm42* by RPA. Dashed line = NPV, or the probability that a sample did not contain macrolide-resistant bacteria, if macrolide ARGs were not detected using RPA. The y-axis dotted line is a theoretical PPV or NPV of 75%, denoting the pretest probabilities of macrolide resistance (x-axis dotted lines) used as theoretical levels at which to trust that RPA will correctly identify a sample with (PPV) or without (NPV) macrolide ARGs at least 75% of the time. The first vertical dotted line on the x-axis is the maximum prevalence at which the NPV >75%. The second vertical dotted line on the x-axis is the minimum prevalence at which the PPV >75% the detection of *msrE-mphE* and/or *erm42* by RPA.

Using the estimated results of the Bayesian model to inform the calculation of PPVs and NPVs, the clinical sensitivity of RPA was 20.5% and specificity was 93.5% for the detection of macrolide resistance. The prior probability of macrolide resistance was 8.8%, based on the prevalence of macrolide ARGs (i.e., total RPA-macrolide-ARG-positive samples divided by the total samples within the testing strategy; 95 of 1,076). Given this information, the PPV, or probability of phenotypic macrolide resistance in samples that tested positive for macrolide ARGs using RPA, was 23% (**Fig. 2**). Using the same information, the NPV, or proportion of samples susceptible to macrolides given a negative RPA test result, was 92% (Fig. 2). Theoretically, the probability that a positive result from RPA would correctly identify a sample with macrolide-resistance genes was at least 75% when the pretest probability of resistance was >49%. In contrast, a negative RPA result would correctly identify a sample without macrolide-resistance genes 75% of the time when the pretest probability of resistance was <28%.

## Discussion

We used a RPA assay to test >1,000 DNPS samples from fall-placed beef calves collected early in the feeding period, alongside current laboratory test methods employed by a regional commercial veterinary diagnostic laboratory. Although we detected BRD-associated bacterial targets less frequently by RPA than by BC, no difference was found in the detection of *M. bovis* between RPA and rtPCR. However, RPA detected almost 3× more samples containing macrolide-resistance genes *msrE-mphE* or *erm42* than did phenotypic macrolide resistance based on BC-AST. Given that RPA cannot associate the presence of ARGs with a particular organism, some of these detected genes were likely harbored by bacteria unrelated to BRD.^32^ Other common laboratory tests, such as rtPCR and 16S rRNA sequencing, share a similar limitation in linking ARGs to specific pathogens or achieving species-level resolution.^14,38,42,50^

In a search of PubMed, CAB Direct, Scopus, and Google Scholar using the search terms “recombinase polymerase amplification”, “Bayesian latent class”, and “cattle” in all possible fields, we retrieved no reports of BLCMs used to evaluate RPA performance compared with BC, BC-AST, or rtPCR for detecting BRD-related bacterial pathogens and macrolide ARGs in beef calves, suggesting that this analysis has not been reported previously in feedlot cattle. In the absence of a gold standard, BLCMs offer estimates of clinical sensitivity and specificity for each test. In our group of calves, the estimated clinical specificity of RPA did not differ from BC for the detection of *Pasteurellaceae*. Clinical, or diagnostic, specificity reflects the ability of RPA or BC to correctly classify DNPSs that did not contain the associated target of interest. However, the sensitivity of RPA, or its ability to identify samples containing BRD-associated bacteria, was significantly lower than BC. In contrast, the specificity of RPA for *M. bovis* was higher than PCR, whereas the sensitivity did not differ, as indicated by the overlapping CrIs.

Both BC-AST and RPA had low sensitivity for their respective resistance targets (phenotypic macrolide resistance in *M. haemolytica*, *P. multocida*, and/or *H. somni*, and macrolide ARGs [*msrE-mphE* and/or *erm42*]), reflecting a high potential for false negatives. However, the specificity of RPA for detecting macrolide ARGs in *Pasteurellaceae* was lower than that of BC-AST for identifying phenotypic macrolide resistance. The lower specificity of RPA compared with BC-AST likely reflects detection of ARGs in non-target, commensal, or environmental bacteria.^32^ Additionally, discrepancies may have occurred if colonies selected for AST lacked these genes, whereas untested variants within the same sample possessed them. Given that RPA was applied to DNA extracted from whole DNPS samples, RPA could detect these genes regardless of bacterial origin, whereas BC-AST could identify resistance only in the chosen, cultured *Pasteurellaceae* isolates.

The validity of the test performance estimates from the BLCMs was dependent on 2 main assumptions. First, the prevalence of the targeted BRD pathogens or macrolide resistance/ARGs was expected to differ among the cattle population,^33,48^ based on sampling times and the antimicrobial metaphylaxis protocol. In our previous study,^1^ we found differences in the prevalence of BRD-related bacteria and phenotypic AMR among various sampling times and metaphylaxis protocols. For example, recovery of *M. haemolytica* from cattle treated with TUL decreased from 1 DOF to 13 DOF, then increased from 13 DOF to 36 DOF, compared with cattle receiving oxytetracycline, in which the recovery of *M. haemolytica* from DNPSs decreased from 1 DOF to 36 DOF.^1^ Several other studies have also noted shifts in the distribution of nasopharyngeal microbiota and prevalence of ARGs over time and between metaphylaxis protocols.^38,60,61^

Differences in prevalence among the populations designated in the BLCM can also be limited by the overall prevalence of the targets of interest in a study population. The sensitivity estimates from the BLCM for macrolide resistance in our study were likely limited by the low prevalence (3%) of *Pasteurellaceae* isolates with phenotypic macrolide resistance.^1^ In contrast, all other key outcomes of interest were detected in at least 10% of our study samples. Prevalence plays a crucial role in determining the precision and accuracy of sensitivity and specificity estimates when using BLCMs. When prevalence is extremely low or very high, data may be insufficient to reliably assess the performance of a laboratory test.^34^

The second key assumption of BLCMs is conditional independence among tests.^33,38^ In our study, BC-AST and RPA were considered independent for detecting BRD-associated bacteria and macrolide resistance. However, covariance terms were incorporated into the models for *M. bovis*, to account for the expected correlation between RPA and PCR, as both are nucleic acid–based assays. An advantage of BLCMs compared with other latent class models for estimation of test performance, in the absence of a gold standard, is their ability to include covariance terms to account for correlation between tests, assuming that the model degrees of freedom are sufficient to allow for estimation of additional parameters.^48^

Samples that were RPA-positive for any of *M. haemolytica*, *P. multocida*, or *H. somni* were also tested for 2 ICE variants. Studies have reported ICE-mediated conjugative transfer and its contribution to AMR,^12,18,31,44^ but most were limited by small sample sizes or laboratory-based conjugation models.^12,22,25,31,44^ Again, as above, our work here has not been reported previously as a large-scale field application to assess whether ICE detection by RPA is associated with phenotypic AMR in respiratory bacteria from feedlot calves.

The ICE variant targets (ICE*tnpA*, ICE*ebrB*) of the RPA assay^24,25^ have been shown to be associated with BRD bacteria.^8,31,44^ Although the ARGs harbored by *Pasteurellaceae-*associated ICEs can differ,^11^ both variants include *tetH* (which encodes a tetracycline efflux pump) along with either transposase A (*tnpA*) for ICE*tnpA*, or the highly conserved multidrug efflux transporter gene (*ebrB*) of the ICE*ebrB* variant.^12,22,24,25^ We found that RPA identification of either variant was associated with more frequent detection of phenotypic resistance, including tetracycline and macrolide resistance, as well as multiclass resistance in samples from both healthy and sick calves. We specifically examined the association between ICE detection and macrolide resistance based on earlier reports that *Pasteurellaceae*-associated ICEs harbor macrolide ARGs, including *msrE*, *mphE*, and *erm42*, among others.^11,22,51^ Also, as expected, given that *tetH* is a component of ICE*tnpA* and ICE*ebrB*, the presence of these ICEs was strongly associated with tetracycline resistance.

Although *tetH* was associated with tetracycline-resistance phenotype, concordance was not perfect. However, the *tetH* gene is only one determinant conferring tetracycline resistance,^13,57^ and phenotypic outcomes can vary depending on gene expression and host context. Furthermore, AST reflects resistance in a single cultured isolate, whereas RPA detects genetic targets across total DNA in a sample. Therefore, discrepancies likely occurred when bacterial species beyond the scope of AST carried alternate tetracycline genes or ICE variants within a sample.

Studies have identified significant associations between calf-level and pen-level BC-AST and metagenomic results from DNPS samples collected at 13 DOF and subsequent phenotypic AMR and BRD treatments, specifically involving the recovery of *M. haemolytica*, *P. multocida*, or *H. somni* with concurrent tetracycline and macrolide resistance.^2,3^ Although the agreement between RPA detection of ARGs and phenotypic resistance in our study was very slight, the findings from other studies suggested that BC-AST results and identification of bacteria and ARGs from calf respiratory samples obtained at ~2 wk on feed could help predict the future risk of BRD.^2^ With evidence of AMR, these results could then be used to inform antimicrobial treatment protocols to reduce further selection for resistance.^2,3^

One methodologic and protocol-related limitation of our study was low RPA sensitivity for bacterial detection, consistent with previous reports, likely leading to false negatives and missed downstream detection of ICE*tnpA*, ICE*ebrB*, *msrE-mphE*, and *erm42*.^25,32^ Future assay optimization could target improved multiplex performance by minimizing competitive inhibition among targets or depletion of reagents by the internal amplification control.^25,26^ Testing all samples for ICE*tnpA*, ICE*ebrB*, *msrE-mphE*, and *erm42* was not practical in our study because of cost; those results also could have complicated interpretation, given that these resistance determinants may occur in non-BRD bacteria.^18,44^

Second, the BLCM analysis was limited to evaluating assays for *M. haemolytica* that were not completely comparable. The RPA assay for *M. haemolytica* detection was intentionally designed to be specific to serotypes 1 and 6, which are more frequently associated with clinical BRD in cattle than serotype 2.^24,45^ Consequently, RPA sensitivity for detecting ICEs or macrolide ARGs may have been underestimated by excluding serotype 2.

A third limitation arose from comparing phenotypic macrolide resistance from single colonies of *M. haemolytica*, *P. multocida*, or *H. somni* to detection of macrolide ARGs in whole-sample DNA. Although the overall prevalence of macrolide resistance was low and occurred primarily in *M. haemolytica*, individual cattle can harbor multiple, genetic variants of the same bacterial species.^20,21^ If isolates of differing genotypes were recovered, a macrolide-resistant isolate could have been overlooked.^20,21^ However, a 2022 investigation also reported limited phenotypic variability among multiple *M. haemolytica* isolates from the same animal, supporting the notion that a single isolate could adequately represent overall susceptibility.^21^ Although broader colony testing might have improved sensitivity or apparent specificity of RPA results, we intentionally designed our study to align with veterinary diagnostic laboratory practices, in which only one isolate per species typically would be subjected to AST. Given cost and logistics, testing multiple isolates per sample remains impractical in routine veterinary testing.

RPA is inherently limited to detecting predefined nucleic acid targets. Although the ARGs included in our study (*msrE*, *mphE*, *erm42*) are recognized determinants of macrolide resistance in BRD-related bacteria,^5,43,62^ they represent a limited number of the potential genetic and epigenetic mechanisms that confer macrolide resistance. Hence, perfect agreement would not be expected, even when testing individual colonies. Beyond these targets, 23S rRNA point mutations can alter the ribosomal macrolide binding site. Furthermore, genes such as *estT* and *estX* confer resistance through macrolide hydrolysis.^28,29,49,53^ Consequently, reliance on target-based ARG detection constrains identification of emerging or previously undescribed resistance mechanisms contributing to resistance. In contrast, AST captures phenotypic resistance mediated by a wide range of mechanisms. However, it does not reveal the underlying genetic determinants, whose expression may vary or be repressed.^13,18^ Furthermore, the range of dilutions used in MIC panels differ across assays, and CLSI breakpoints are not established for all bacterial BRD pathogens.^5,7,23^ For tilmicosin, a breakpoint is only available for *M. haemolytica.*^23^ These inconsistencies affect MIC interpretation and can complicate assessment of expressed resistance across bacterial species. As a target-specific method, RPA may be better suited as a rapid tool to indicate the presence of some important macrolide ARGs and ICEs in a sample, where detection of ICEs suggests an increased risk of resistance prompting further confirmatory testing to guide AMU decisions.

Limited sample volume constrained the number of RPA reactions possible per sample in our investigation, resulting in incomplete ICE and ARG testing. To minimize selection bias, the prevalence estimates from tested samples were extrapolated to untested ones, correcting for potential misclassification resulting from an insufficient volume of extracted sample DNA. This correction was necessary to avoid the misclassification of samples with insufficient volume as “negative.” However, low DNA yields from some of the DNPS samples from both healthy and sick cattle highlights the challenge of obtaining sufficient template DNA for additional assay targets. The restriction of the T16-ISO platform to 2 gene targets per assay further limited multiplexing capacity, increasing both the overall number of reactions and the total amount of DNA required.

Finally, operational challenges also affected throughput feasibility. Despite the rapid runtime of individual RPA reactions, processing thousands of samples across multiple targets on a single T16-ISO unit was both time- and labor-intensive. Each RPA assay required ~15 min for setup and 30 min of runtime, translating to 90–180 min per full set. Testing DNPS samples from a single 100-calf pen required at least 15 complete runs. Combined with machine maintenance demands, our analysis of these reaction constraints suggested that although RPA offers speed and simplicity for small-scale or targeted testing, it is not yet practical for making large-scale, high-throughput point-of-care decisions on AMU.

Through this study, we demonstrated the successful application of RPA in a large cohort of commercially sourced, fall-placed calves to detect bacterial pathogens and AMR determinants relevant to BRD management. Although RPA showed promise, its estimated sensitivity for *M. haemolytica*, *P. multocida*, and *H. somni* was notably lower than that of BC, likely contributing to the low estimated sensitivity of RPA for macrolide ARG detection. RPA specificity for macrolide ARGs was also lower than BC-AST, suggesting detection of ARGs in non-target organisms.

Importantly, RPA-based identification of ICE gene pairs containing *tetH* was associated with an increased likelihood of concurrent phenotypic resistance to tetracyclines, macrolides, and multiclass resistance. Our findings extended the results of published, small-scale, cross-sectional or case-based molecular studies, and are a meaningful step toward understanding the utility of ICE detection in estimating AMR at the population level. However, RPA and other target-based tests cannot directly link BRD pathogens to specific ARGs, limiting their standalone utility in guiding AMU decisions. Hence, when used alone, RPA was not sufficient to inform AMU treatment strategies. However, with continued target optimization and a minimal time to results, RPA could potentially be used to address focused questions, as part of a comprehensive diagnostic plan.

## Supplemental Material

sj-pdf-1-vdi-10.1177_10406387261423941 – Supplemental material for Evaluation of recombinase polymerase amplification assays for targeted detection of bovine respiratory disease bacterial pathogens and antimicrobial-resistance genes in feedlot calves Supplemental material, sj-pdf-1-vdi-10.1177_10406387261423941 for Evaluation of recombinase polymerase amplification assays for targeted detection of bovine respiratory disease bacterial pathogens and antimicrobial-resistance genes in feedlot calves by Tara Funk, Lianne McLeod, Rahat Zaheer, Curtis Claassen, Christina Yevtushenko, Cheyenne Conrad, Jennifer Abi Younes, Morgan Lehmann, Sheryl Gow, Bruce Wobeser, Simon J. G. Otto, Cheryl Waldner and Tim McAllister in Journal of Veterinary Diagnostic Investigation
