# Supplementary material for: Evaluation of recombinase polymerase amplification assays for targeted detection of bovine respiratory disease bacterial pathogens and antimicrobial-resistance genes in feedlot calves
Source: J Vet Diagn Invest. 2026 Mar 4:10406387261423941. Online ahead of print. doi: 10.1177/10406387261423941 (PMC12962989; doi:10.1177/10406387261423941)
Supplement: sj-pdf-1-vdi-10.1177_10406387261423941 – Supplemental material for Evaluation of recombinase polymerase amplification assays for targeted detection of bovine respiratory disease bacterial pathogens and antimicrobial-resistance genes in feedlot calves [file sj-pdf-1-vdi-10.1177_10406387261423941.pdf]

Funk T, et al. Evaluation of recombinase polymerase amplification assays for targeted detection of bovine respiratory disease bacterial pathogens and antimicrobial-resistance genes in feedlot calves

Samples collected from calves treated for bovine respiratory disease

BRD-related bacterial pathogen detection

The prevalence of bacterial pathogens detected with bacterial culture was higher than recombinase polymerase amplification (RPA) for samples collected from 74 calves at the time of BRD treatment (**Suppl. Table 1**). RPA and bacterial culture had slight-to-moderate agreement for detection of BRD-associated bacterial pathogens—*M. haemolytica* $\kappa = 0.25$ (95% CI [0.09, 0.42]), *P. multocida* $\kappa = 0.18$ (95% CI [0.01, 0.35]), and *H. somni* $\kappa = 0.54$ (95% CI [0.32, 0.76]). Kappa could not be calculated between RPA and PCR for *M. bovis* detection, as no positive samples were detected by PCR.

Supplemental Table 1. Bacteria associated with bovine respiratory disease detected by recombinase polymerase amplification (RPA), PCR, and culture of deep nasopharyngeal swab samples – samples from 74 calves immediately before first treatment for respiratory disease.

	Count of positive samples (%)		
Species	RPA	Culture	PCR
Mannheimia haemolytica (RPA: serotypes 1, 6)	20 (27)	47 (64)	NA
Pasteurella multocida	4 (5.4)	27 (37)	NA
Histophilus somni	20 (27)	22 (30)	NA
Mycoplasmopsis bovis	8 (11)	NA	0 (0.0)

NA = not applicable; RPA = recombinase polymerase amplification.

Tetracycline resistance and ICE detection

Thirty-seven samples from sick calves were positive for *M. haemolytica*, *P. multocida*, and/or *H. somni* by RPA. Two pathogen-positive samples did not contain sufficient volume for ICE testing. Variants ICE*tnpA* and ICE*ebrB* were more frequently identified individually than together in the remaining 35 samples (**Suppl. Table 2**).

Using bacterial culture, 91% of the 74 samples were positive for *M. haemolytica*, *P. multocida*, and/or *H. somni.* Of these pathogen-positive samples, 20% contained at least one bacterial isolate that was resistant to tetracycline based on AST.

RPA detection of ICE variants containing *tetH* and phenotypic tetracycline resistance using AST demonstrated fair agreement ($\kappa = 0.37$; 95% CI [0.10, 0.64]). When applied to the whole testing strategy, the presence of ICEs was a very strong predictor of tetracycline resistance (OR 15, $p < 0.001$; **Suppl. Table 3**).

Supplemental Table 2. Prevalence of integrative and conjugative element variants identified by recombinase polymerase amplification (RPA), and phenotypic tetracycline resistance of *Mannheimia haemolytica*, *Pasteurella multocida*, or *Histophilus somni* isolated from deep nasopharyngeal swabs collected from calves at first treatment for respiratory disease.

Tetracycline resistance and ICE gene combination	No. of positive samples (*n* = 35)‡	% positive samples, by the no. of samples tested using RPA (*n* = 35)	Estimated no. of positive samples based on eligible samples for RPA testing (*n* = 37)	% positive, by the no. of samples tested using culture (*n* = 67)	% positive samples, based on the testing strategy (*n* = 74)
AST results*					
Phenotypic tetracycline resistance	15	NA	NA	22	20
RPA results†					
ICE*tnpA* only	9	26	10	NA	14
ICE*ebrB* only	12	34	13	NA	18
ICE*tnpA* and ICE*ebrB*	1	2.9	1	NA	1.4
Total ICE-positive samples	22	63	24	NA	32

AST = antimicrobial susceptibility testing, specifically for expressed tetracycline resistance; ICE = integrative and conjugative element, including variants ICEtnpA (tetH_tnpA target sequence) and ICEebrB (tetH_ebrB target sequence), which both contain the tetracycline resistance gene, tetH; NA = not applicable.

* Samples tested by AST were positive for any of *M. haemolytica*, *P. multocida*, or *H. somni*.

† Only samples that were RPA-positive for at least one of *M. haemolytica*, *P. multocida*, or *H. somni* were eligible for testing for ICEtnpA and ICEebrB.

‡ Total no. of samples eligible for testing with sufficient volume.

Supplemental Table 3. Association between detection of integrative and conjugative elements and phenotypic resistance to tetracycline, macrolides, or any antimicrobial drug tested, or multiclass resistance in *Mannheimia haemolytica*, *Pasteurella multocida*, or *Histophilus somni* isolated from deep nasopharyngeal swabs from calves at first treatment for respiratory disease.

Phenotypic resistance variant	No.	Odds ratio	95% CI	*p* value
Any antimicrobial drug (excluding TET)	71	3.3	[0.99, 11]	0.052
Any antimicrobial drug (including TET)	71	8.2	[3.6, 19]	<0.001
Macrolides (GAM, TIL, TILD, TUL)	13	2.5	[1.3, 4.8]	0.005
Multiclass resistance	13	2.5	[1.3, 4.8]	0.005
Tetracycline	58	15	[12, 19]	<0.001

The generalized estimating equation models developed were applied using the whole sample testing strategy, adjusting for clustering of resistance by pen, and accounting for time point and the type of metaphylaxis (OTC or TUL) administered as independent variables with fixed effects. Antimicrobials included on the AST panel included: ampicillin (AMP), ceftiofur (TIO), danofloxacin (DANO), enrofloxacin (ENR), florfenicol (FLOR), gamithromycin (GAM), penicillin (PEN), spectinomycin (SPECT), tetracycline (TET), tildipirosin (TILD), tilmicosin (TIL), and tulathromycin (TUL). Note: the MIC distribution of TIL was only interpreted for isolated *M. haemolytica* colonies, because Clinical and Laboratory Standards Institute recommended breakpoints are not available for *P. multocida* and *H. somni*.

Macrolide resistance and antimicrobial resistance gene detection

Accounting for 3 more samples with insufficient volume following ICE testing, macrolide ARGs *msrE-mphE* and *erm42* were most often identified together in the samples tested (38%; **Suppl. Table 4**). Most (97%) samples contained bacteria that were susceptible to all macrolide antimicrobial drugs tested (Suppl. Table 4).

RPA and AST did not agree ($\kappa < 0.001$) for detection of macrolide ARGs and phenotypic macrolide resistance in the tested samples ($n = 32$). Of those samples that tested positive for *msrE-mphE* and/or *erm42*, none of the isolated BRD bacteria were resistant to macrolides. However, at the calf level, the odds of detecting phenotypic macrolide resistance by AST were 2.5 times higher ($p < 0.001$) given the presence of ICEs (Suppl. Table 3).

The detection of ICEs was also associated with the detection of multiclass resistance or any phenotypic resistance to at least one of the antimicrobial drugs tested (Suppl. Table 3).

Supplemental Table 4. Prevalence of phenotypic macrolide resistance detection using antimicrobial susceptibility testing (AST) of *Mannheimia haemolytica*, *Pasteurella multocida*, or *Histophilus somni* or macrolide antimicrobial resistance genes (ARGs) detected by recombinase polymerase amplification (RPA) of bacteria of interest isolated from deep nasopharyngeal swabs for samples from calves at first treatment for respiratory disease.

Macrolide resistance variation (ARG or phenotypic)	Count of positive samples (*n* = 32)§	% positive, by the number of samples tested using RPA (*n* = 32)	Estimated count of positive samples based on eligible samples for RPA testing (*n* = 37)	% positive, by the number of samples tested using culture (*n* = 74)	% positive samples, based on the testing strategy (*n* = 74)
AST results*					
Phenotypic macrolide resistance†	2	NA	NA	2.7	2.7
RPA results‡					
msrE-mphE only	6	19	7	NA	9.5
erm42 only	5	16	6	NA	8.1
msrE-mphE and *erm42*	12	38	14	NA	19
Total macrolide ARG-positive samples	23	72	27	NA	37

NA = not applicable.

* Samples tested by AST were positive for any of *M. haemolytica*, *P. multocida*, or *H. somni*.

† Phenotypic macrolide resistance was defined by the presence of resistance to gamithromycin, tilmicosin, tildipirosin, and/or tulathromycin following AST.

‡ Only samples that were RPA-positive for at least one of *M. haemolytica*, *P. multocida*, or *H. somni* were eligible for testing for macrolide ARGs.

§ Total no. of samples eligible for testing with sufficient volume.
